# Abdominoperineal Resection in a Male-to-Female Transgender Patient With Anal Cancer and Neovaginal Resection: A Case Report

**DOI:** 10.7759/cureus.86840

**Published:** 2025-06-27

**Authors:** Yosor Fiesal, Hayim Gilshtein

**Affiliations:** 1 General Surgery, Rambam Medical Center, Haifa, ISR

**Keywords:** abdominoperineal resection, anal canal cancer, case report, gender-affirming surgery, hpv, neovagina, sigmoid vaginoplasty, transgender health

## Abstract

Anal cancer is a rare malignancy with rising incidence, largely attributed to human papillomavirus (HPV) infection. Transgender women (TGW) are particularly vulnerable to HPV-related cancers due to higher rates of HPV acquisition, and gender-affirming surgeries, such as vaginoplasty, introduce unique anatomical challenges in management.

We present the case of a 58-year-old TGW with a history of gender-affirming hormonal therapy and vaginoplasty using a bowel segment, who presented with anal symptoms and was diagnosed with well-differentiated, HPV p16-positive squamous cell carcinoma. Staging revealed a locally advanced tumor without metastasis. She underwent chemoradiation with mitomycin C (MMC) and 5-fluorouracil (5-FU), achieving an initial complete response, but positron emission tomography (PET) imaging a year later detected recurrence. A multidisciplinary team (MDT) recommended abdominoperineal resection (APR); however, intraoperative findings revealed a neovagina composed of sigmoid colon, precluding rectal resection alone, and the surgery was initially aborted. A month later, a combined APR with neovagina resection was performed, and pathology showed complete tumor response. The patient recovered well postoperatively.

This case underscores the importance of obtaining detailed surgical histories and thoroughly reviewing preoperative imaging in transgender patients. Clinicians must be prepared for unexpected anatomical variations that require surgical adaptation and consider the psychosocial impact of neovaginal loss. Ultimately, comprehensive, multidisciplinary care is essential for optimizing the oncological and functional outcomes of transgender patients with anal cancer.

## Introduction

Anal cancer, although uncommon, has shown a rising incidence over the past three decades [1], with approximately 90% of cases attributed to anal human papillomavirus (HPV) infection [2]. Major risk factors for HPV acquisition - and subsequent anal cancer development - include smoking, multiple sexual partners, receptive anal intercourse, and a history of sexually transmitted infections (STIs), including human immunodeficiency virus (HIV) [2]. Notably, these risk factors are more prevalent in male-to-female transgender women (TGW) than in the general population, rendering this group particularly vulnerable to anal cancer [3].

The primary aim of treatment in anal cancer is to achieve cure with locoregional control, preservation of anal function, and the best possible quality of life. Combinations of mitomycin C (MMC) and 5-fluorouracil (5-FU)-based chemoradiotherapy (CRT) have been established as the standard of care, leading to complete tumor regression in 80%-90% of patients [4]. If pathological evidence of recurrence is diagnosed, surgical management by abdominoperineal resection (APR) is recommended. A salvage APR is required in up to 30% of cases, either due to inadequate response to primary treatment or subsequent tumor regrowth [5].

The prevalence of transgender people varies around the world; North America has been found to have higher prevalence, while South Asia, Sub-Saharan Africa, and the Middle East have less data [6]. As a result of advocacy efforts, media representation, and cultural changes, transgender issues have gained more attention and awareness, which has improved estimates of the transgender population and led to more inclusive data collection methods [7,8].

The health disparities that transgender people experience - such as greater rates of mental health problems, substance use disorders, and chronic diseases than cisgender populations - are becoming more widely recognized [6-8]. Calls for better data collection, standardized inquiries to identify transgender respondents, and focused public health measures to address these inequities have been sparked by this understanding [7,8].

Cancer risk is an emerging priority in transgender health research, with particular attention to the elevated risks of HPV and associated malignancies in TGW. TGW are more likely to engage in receptive anal intercourse, which is a high-risk route for transmission of both HPV and HIV [9]. Despite limited data, studies suggest that TGW have higher rates of HPV infection, which can lead to anal dysplasia and, ultimately, cancer. For instance, a study of a small cohort of TGW with HIV found that 91% had abnormal anal cytology, and 91% had abnormal histology [10].

The most common genital gender-affirming surgery for TGW is vaginoplasty, with penile inversion vaginoplasty being the gold standard technique. This procedure involves the creation of a neovagina using the penile and scrotal skin [11]. Alternative techniques include peritoneal vaginoplasty and intestinal vaginoplasty - especially the sigma - which may be indicated in specific cases [11,12]. These anatomical changes must be carefully considered when managing anal cancer or other pelvic cancers. Surgical interventions like APR, required for the treatment of anal cancer, may be particularly challenging, as they often necessitate resection of neovaginal tissue [12].

This case report presents the complex surgical management of anal cancer in a TGW, highlighting the need for a multidisciplinary, individualized approach to care in this population.

## Case presentation

A 58-year-old patient underwent gender-affirming hormonal and surgical treatments at the age of 38. Following hormone replacement therapy (HRT), she underwent a penile inversion vaginoplasty performed in England, utilizing her own penile tissue. Six years later, she opted for a second procedure in Thailand to deepen the vaginal canal, during which a neovagina was created. According to the patient, a “bowel segment” was used, probably the small bowel.

More than 12 years after her gender-affirming surgeries, the patient presented to our clinic in June 2024 with new-onset anal pain, rectal bleeding, and intermittent tenesmus. Her significant past medical history included long-term gender-affirming hormonal therapy, and no previous malignancies. She denied previous anal surgeries, aside from the gender-affirming procedures described above.

On physical examination, she was afebrile with stable vital signs. Inspection revealed mild perianal erythema and external scarring consistent with prior surgery, but no obvious masses or ulcerations. Digital rectal examination noted tenderness, with mild narrowing at the anal verge, but no palpable mass. There was no inguinal lymphadenopathy.

Baseline laboratory evaluation revealed a hemoglobin of 10 g/dL, white blood cell count of 8,200 cells/μL, and platelets within normal limits. HIV testing was negative. Additional STI screening was unremarkable.

Colonoscopy demonstrated internal hemorrhoids. No additional lesions were identified in the colon. Biopsy of the lesion revealed features consistent with squamous papilloma. Based on these findings, she underwent a hemorrhoidectomy for symptomatic relief and definitive diagnosis. Pathology showed a well-differentiated, HPV p16-positive squamous cell carcinoma.

**Figure 1:**
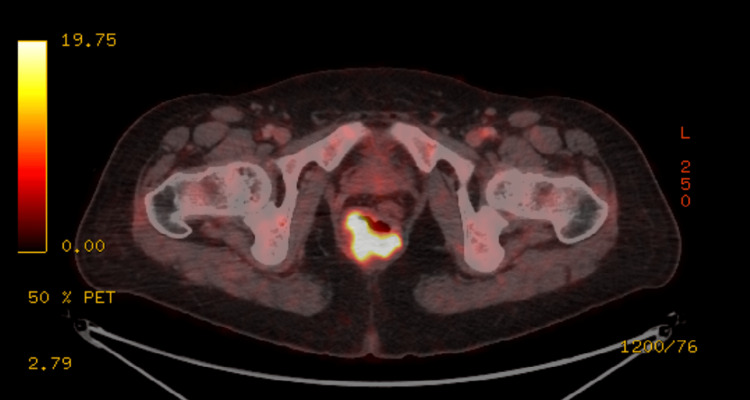
PET-CT demonstrating pathological signal on the right side of the anus and the perianal area PET-CT, positron emission tomography-computed tomography

**Figure 2:**
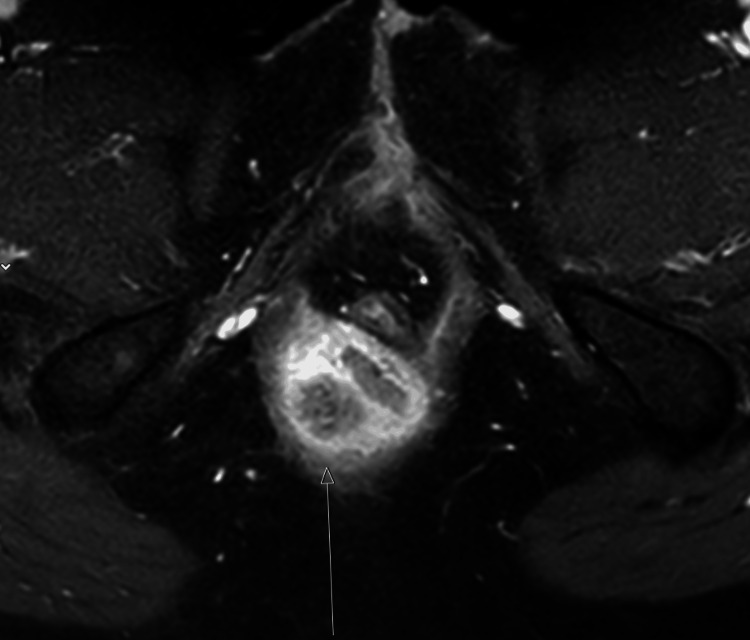
MRI demonstrating tumor on the right side of the anus (arrow) MRI, magnetic resonance imaging

Subsequently, local and systemic staging with pelvic magnetic resonance imaging (MRI) and positron emission tomography-computed tomography (PET-CT) scans confirmed a locally advanced tumor confined to the anal canal, without evidence of metastatic spread (Figures 1-2).

The patient was presented at a multidisciplinary team (MDT) meeting, and following its decision, she received CRT with MMC and 5-FU (MMC + 5-FU) at doses of 10 mg/m² and 1000 mg/m², respectively. External beam radiation therapy (EBRT) was delivered with a total dose of 54 Gy to the anus and 45 Gy to the pelvis and groins. There was no evidence of disease in the first year of follow-up, when a scheduled routine PET scan showed a pathological signal on the right side of the anus and the perianal area, indicating a recurrence of anal cancer. A repeat MDT presentation was initiated, with the recommendation to proceed to APR. The patient was advised, and following a lengthy discussion, informed consent was obtained, and the surgery was scheduled.

In the operating room, during laparoscopic exploration, it was noted that the neovagina was composed of sigmoid colon - a crucial detail that was missing from both the patient’s history and was not addressed when interpreting her imaging studies. This finding rendered the resection of the rectum alone impossible due to their overlapping blood supply, thus making the combined resection of the neovagina mandatory. At this point, given these anatomic considerations, the surgery was aborted.

A detailed explanation was provided to the patient, emphasizing the need for the combined resection of her neovagina with the rectum to ensure complete cancer removal.

**Figure 3:**
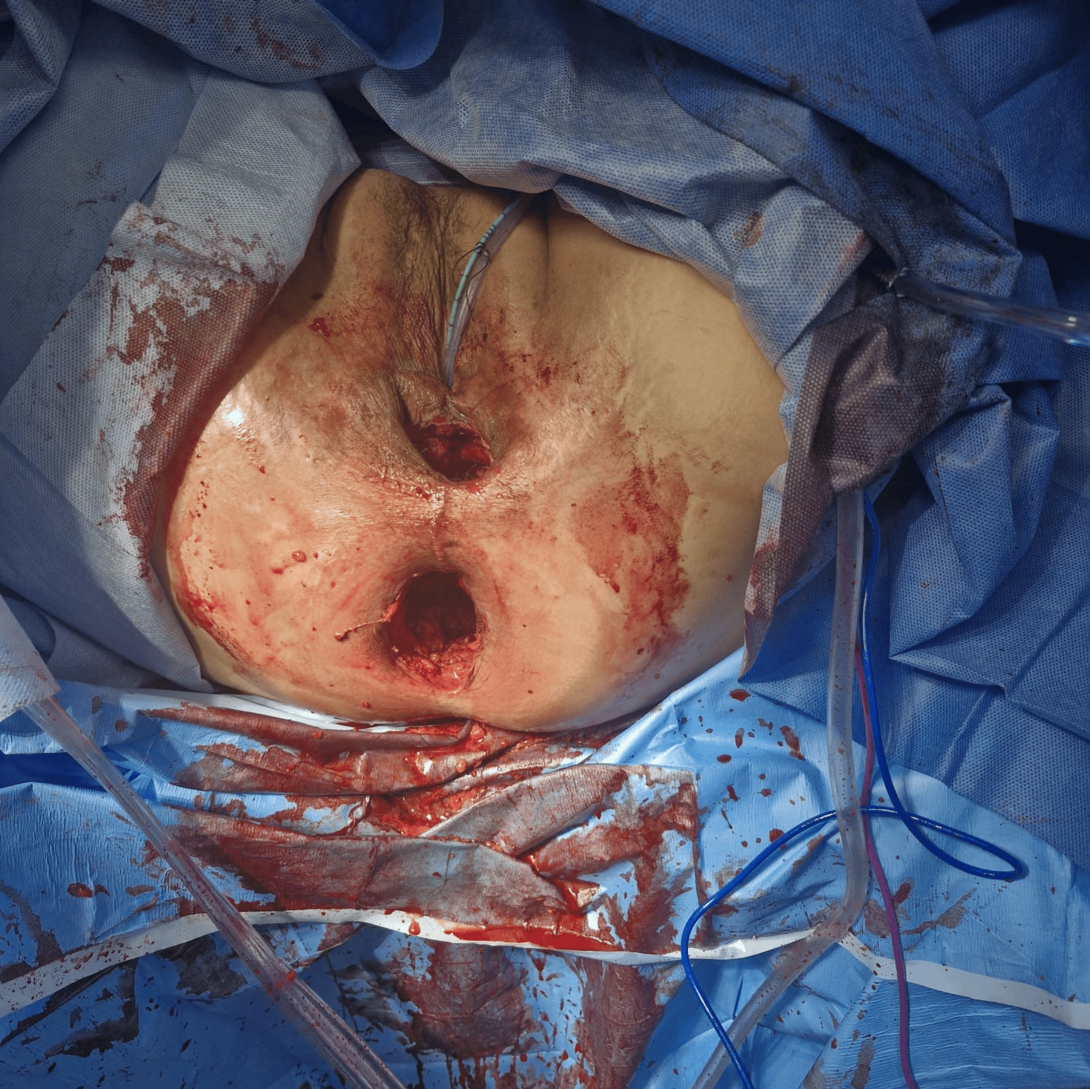
Intraoperative clinical photograph after resecting the anus, the rectum, and the neovagina

The APR surgery was performed a month later, with laparoscopic mobilization of the left colon, residual sigmoid, and rectum; vessel ligation; and a lower midline incision for the demanding pelvic dissection. The surgery was completed with a perineal incision to complete the mobilization and resection of the neovagina and anal canal. A primary closure of the perineal wound combining the orifices of the anal canal and neovagina was performed (Figure 3). Pathology results were as follows: the rectum showed an area measuring 4 cm in largest diameter with erosions and ulceration. No viable tumor cells were seen (complete response). The surgical colonic and radial margins were unremarkable. The neovagina showed squamous epithelium and colonic epithelium with superficial erosions. Four lymph nodes were found, free of tumor.

The patient’s immediate postoperative recovery was uneventful. She remained hospitalized for five days post-surgery, during which time she mobilized with assistance, and her perineal wound was inspected regularly, with no evidence of infection or dehiscence.

Over the first 90 days after discharge, she was seen in the outpatient clinics at two weeks, six weeks, and three months postoperatively. At each visit, the surgical wound was well healed, with minimal pain reported and no signs of delayed healing or infection. She experienced some initial discomfort and mild swelling, which resolved by the third postoperative week.

The patient required regular stoma care education and support, which she managed successfully with help from nursing staff. She reported gradually improving mobility, participated in physiotherapy as recommended, and had no major medical or surgical complications, such as deep vein thrombosis, wound infection, or hospital readmission.

From a psychological standpoint, the patient continued to receive emotional and social support from the hospital’s psycho-oncology and social work teams. She described a period of emotional adjustment following the loss of her neovagina but gradually began to focus on her cancer recovery and overall well-being.

At the three-month follow-up, a repeat clinical and radiological assessment showed no evidence of disease recurrence, excellent wound healing, and a return to independent daily activities. She continues to be followed up at regular intervals in our outpatient clinic.

## Discussion

This case highlights the complex surgical and oncological challenges encountered in treating anal cancer in a TGW who had undergone gender-affirming surgery. The need for specialized, interdisciplinary approaches in the management of transgender patients is highlighted by the growing awareness of transgender health disparities, such as the increased risk of HPV-related cancers [13]. TGW are particularly vulnerable due to the increased prevalence of high-risk sexual behaviors, including receptive anal intercourse and prior STIs, which facilitate HPV transmission and persistence [14]. In line with this, our patient - despite being HIV-negative - presented with HPV-positive anal cancer, a pattern consistent with data from TGW cohorts reporting high rates of abnormal anal cytology and histology [14].

Neovaginal HPV infection and related lesions are quite prevalent in TGW who have had vaginoplasty. Neovaginal HPV prevalence varies between 8.3% and 20%, according to studies. Particularly in sexually active TGW, routine screening for neovaginal HPV and related cytologic abnormalities may be necessary [14]. Additionally, the CDC advises HPV immunization for TGW up until age 26 [15]. Barriers to HPV testing in transgender populations include medical mistrust and stigma. In this case, the lack of routine screening might have caused the diagnosis to be delayed, which emphasizes the necessity of increased awareness and focused prevention measures.

However, there is indeed a paucity of published case reports or series specifically addressing the management of anal cancer in TGW with prior gender-affirming surgeries, particularly those involving intestinal vaginoplasty. Most available literature focuses on cisgender patients, or the technical aspects of gender-affirming surgery, with only rare case reports describing oncologic management in this population [16,17].

The surgical literature confirms that intestinal vaginoplasty, such as sigmoid colon vaginoplasty, introduces unique anatomical considerations. The neovagina, constructed from sigmoid colon, shares its blood supply with the rectum, which can complicate future pelvic oncologic surgery by precluding isolated rectal resection and increasing the risk of compromised vascularity to either the neovagina or the rectum. This anatomical relationship is well described in surgical technique reviews and outcomes studies, which emphasize the need for careful preoperative planning and multidisciplinary management in cases of pelvic malignancy [18,19].

This case highlights the need for a detailed surgical history and comprehensive imaging review to anticipate potential anatomical variations before surgery. The unexpected finding that the patient's neovagina was made of sigmoid colon tissue rather than penile tissue presented one of the biggest surgical obstacles in this case. Some patients have intestinal vaginoplasty for a deeper vaginal canal, but the most popular gender-affirming treatment is penile inversion vaginoplasty [11]. Because the rectum and neovagina share a blood supply, it was not possible to remove the rectum alone due to this anatomical variation, which drastically changed the surgical strategy.

The requirement to remove the neovagina added another layer of complexity, both from a surgical and psychosocial perspective. The neovagina plays an important role in the identification and general well-being of many TGW. Therefore, before undergoing surgery, it was essential to ensure that the patient was well informed of the procedure's ramifications and accepted its necessity through lengthy counseling and collaborative decision-making. Of note, the patient received constant emotional support from our psychologist and social worker throughout her hospitalization. She expressed sadness over the loss of her neovagina, describing it as a symbol of her “identity and struggle against stigmatization.” Despite this, she also conveyed relief and gratitude for the opportunity to “stay alive” and continue her fight against cancer.

In summary, this case underscores the need for better transgender-inclusive medical practice. Healthcare professionals need to understand gender-affirming treatments and how they affect cancer therapy, especially surgeons and oncologists. Furthermore, more research is required to comprehend the long-term functional and oncologic results of APR and other pelvic operations performed on transgender individuals.

## Conclusions

This case highlights the complex surgical and psychosocial considerations involved in managing anal cancer in TGW who have undergone gender-affirming surgeries. Careful attention to surgical history, preoperative imaging, and unique anatomical variations is essential for planning optimal oncologic and functional outcomes. Multidisciplinary collaboration and comprehensive, patient-centered support are crucial, particularly when surgical interventions affect gender-affirming anatomy. As the number of transgender patients continues to grow, increased awareness and expertise in transgender-inclusive medical and surgical care will be vital to address their distinct healthcare needs effectively.
